# Supplementary figures and images for: Optimal transport reveals dynamic gene regulatory networks via gene velocity estimation
Source: PLoS Comput Biol. 2025 May 8;21(5):e1012476. doi: 10.1371/journal.pcbi.1012476 (PMC12118989; doi:10.1371/journal.pcbi.1012476)

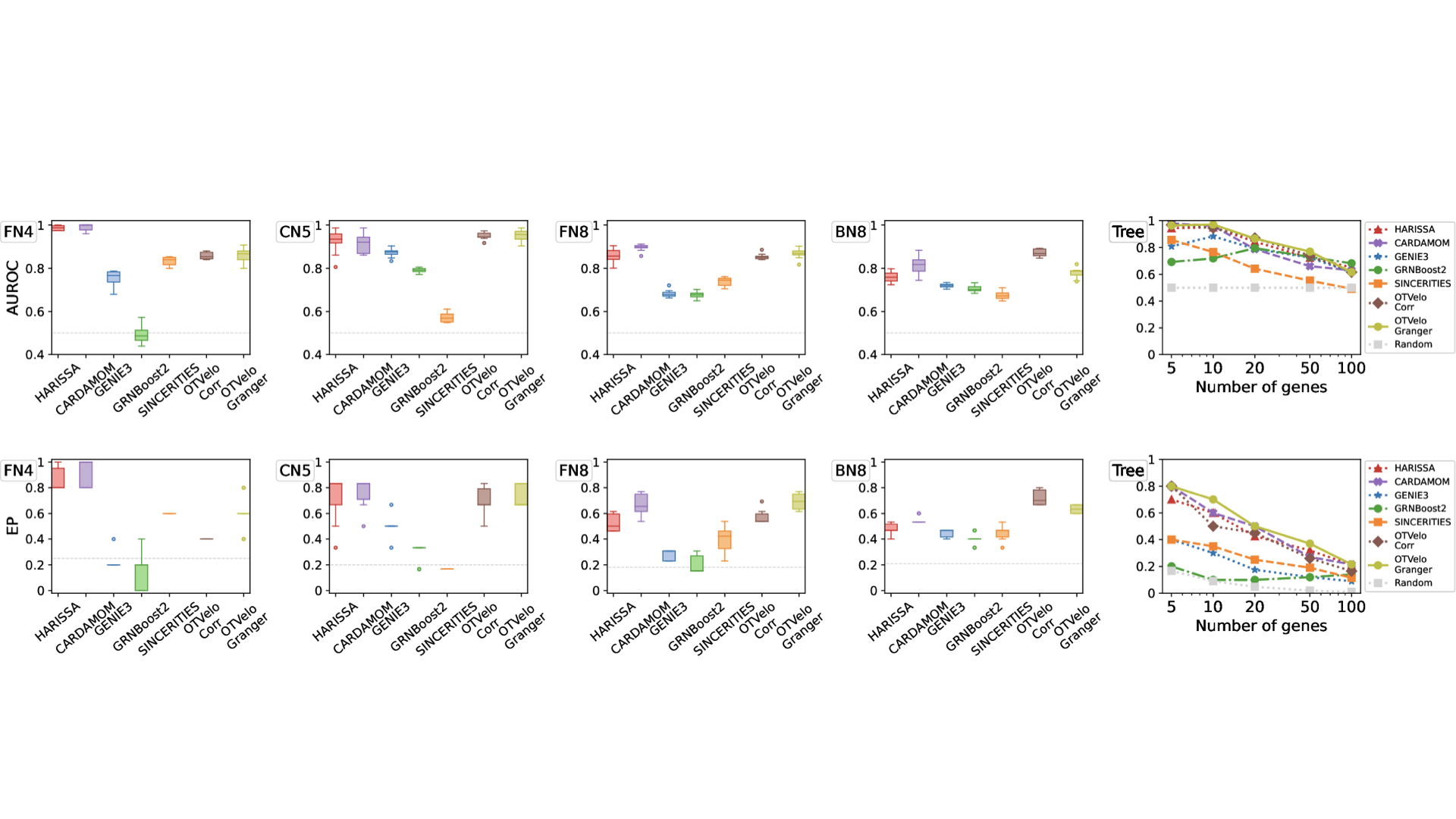

Supplement: S1 Fig — Other performance metrics except AUPRC on datasets simulated from HARISSA [12], including Area under Receiver Operating Characteristic (ROC) curves and early precision as defined by [16]. (TIF) [file pcbi.1012476.s001.tif]

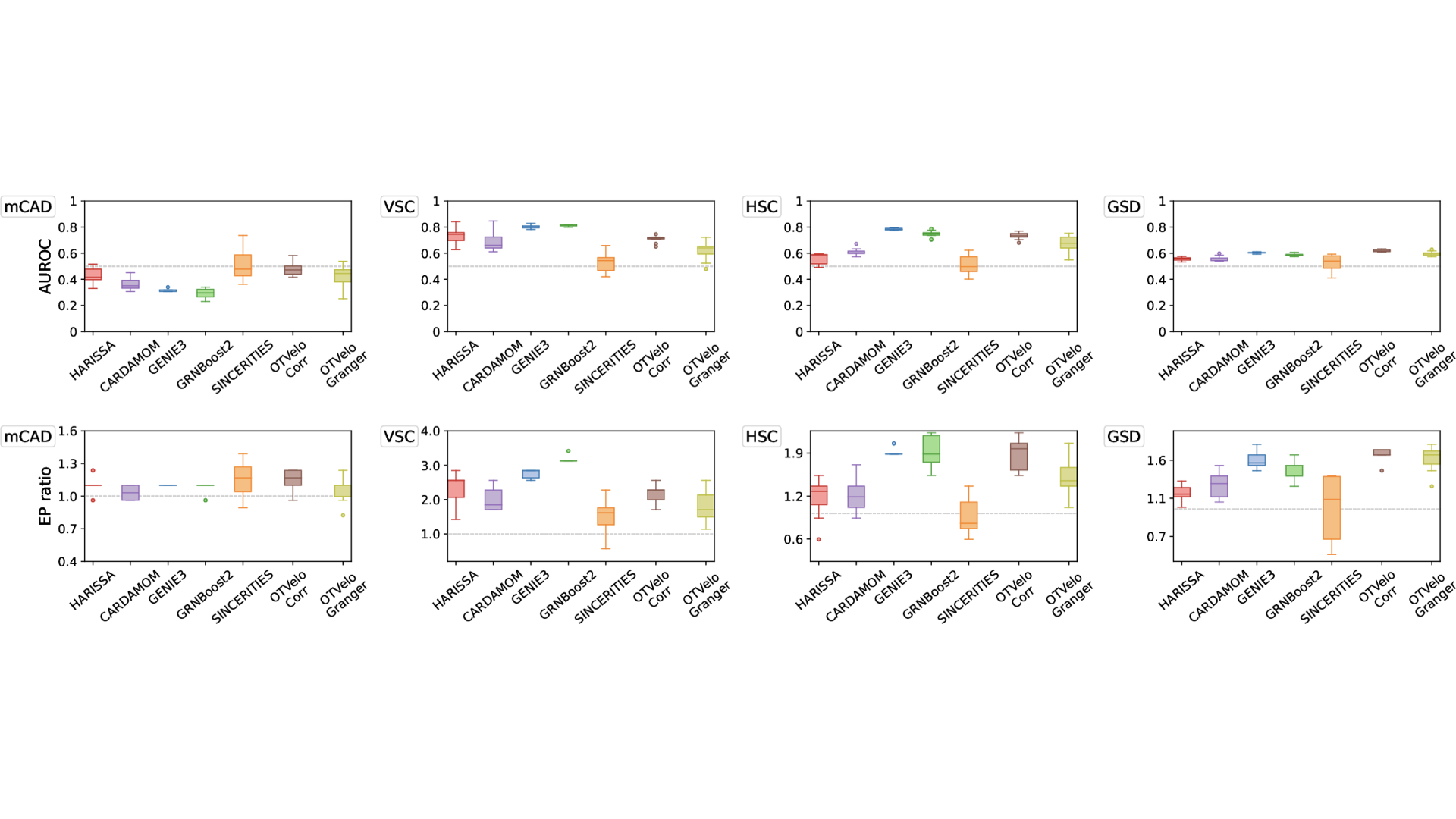

Supplement: S2 Fig — AUROC and EP ratio on datasets simulated from curated networks and BoolODE [16]. The EP ratio equals the early precision value divided by the random baseline, and the value should be greater than one if the performance is better than a random classifier. (TIF) [file pcbi.1012476.s002.tif]

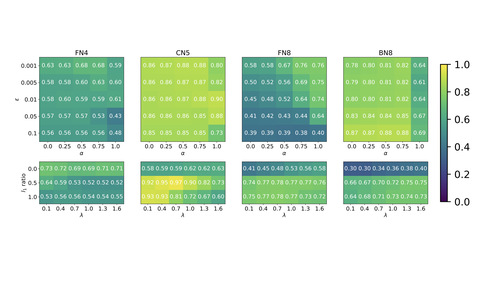

Supplement: S3 Fig — Brighter is better. (TIF) [file pcbi.1012476.s003.tif]

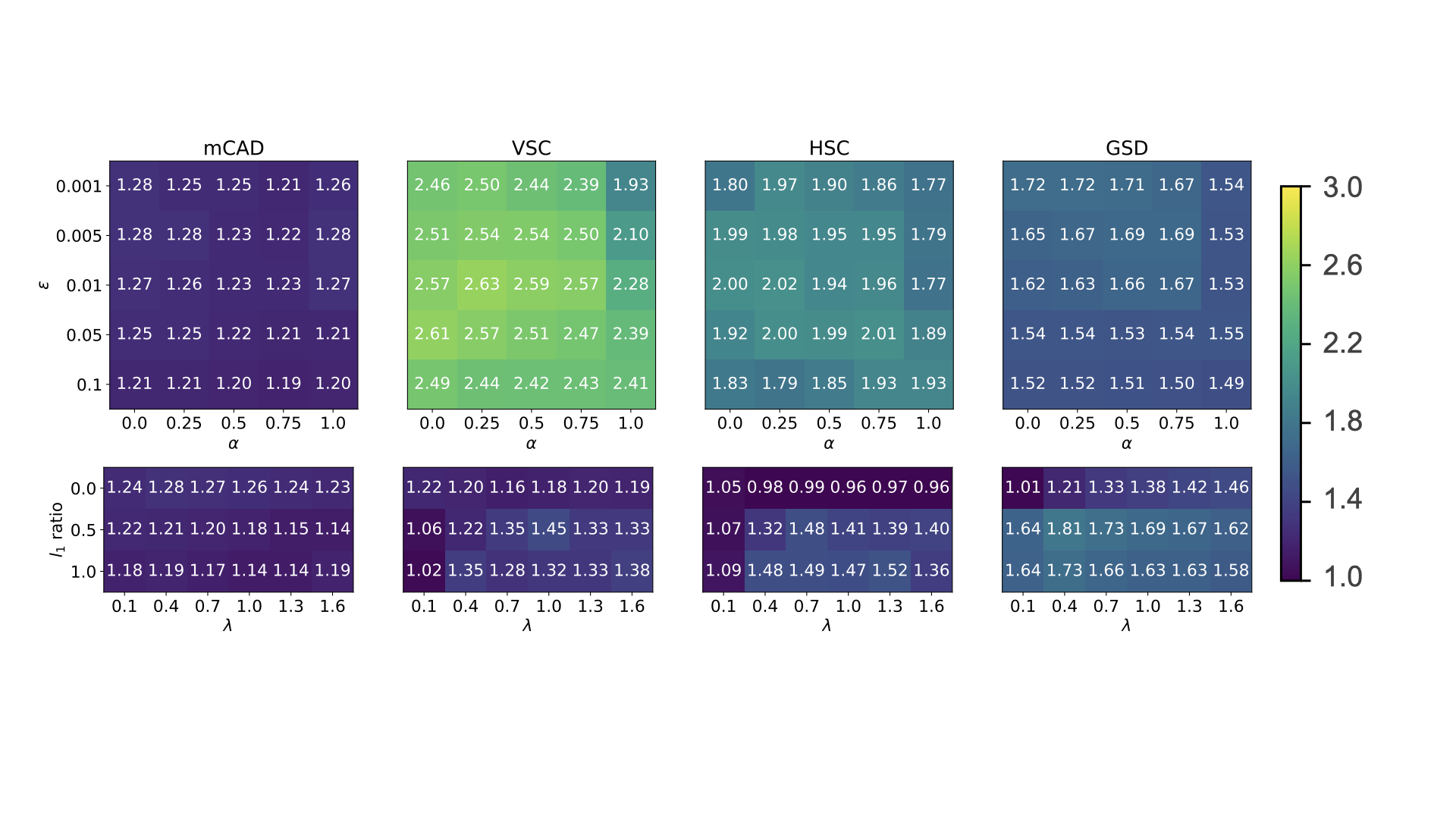

Supplement: S4 Fig — Brighter color is better. (TIF) [file pcbi.1012476.s004.tif]

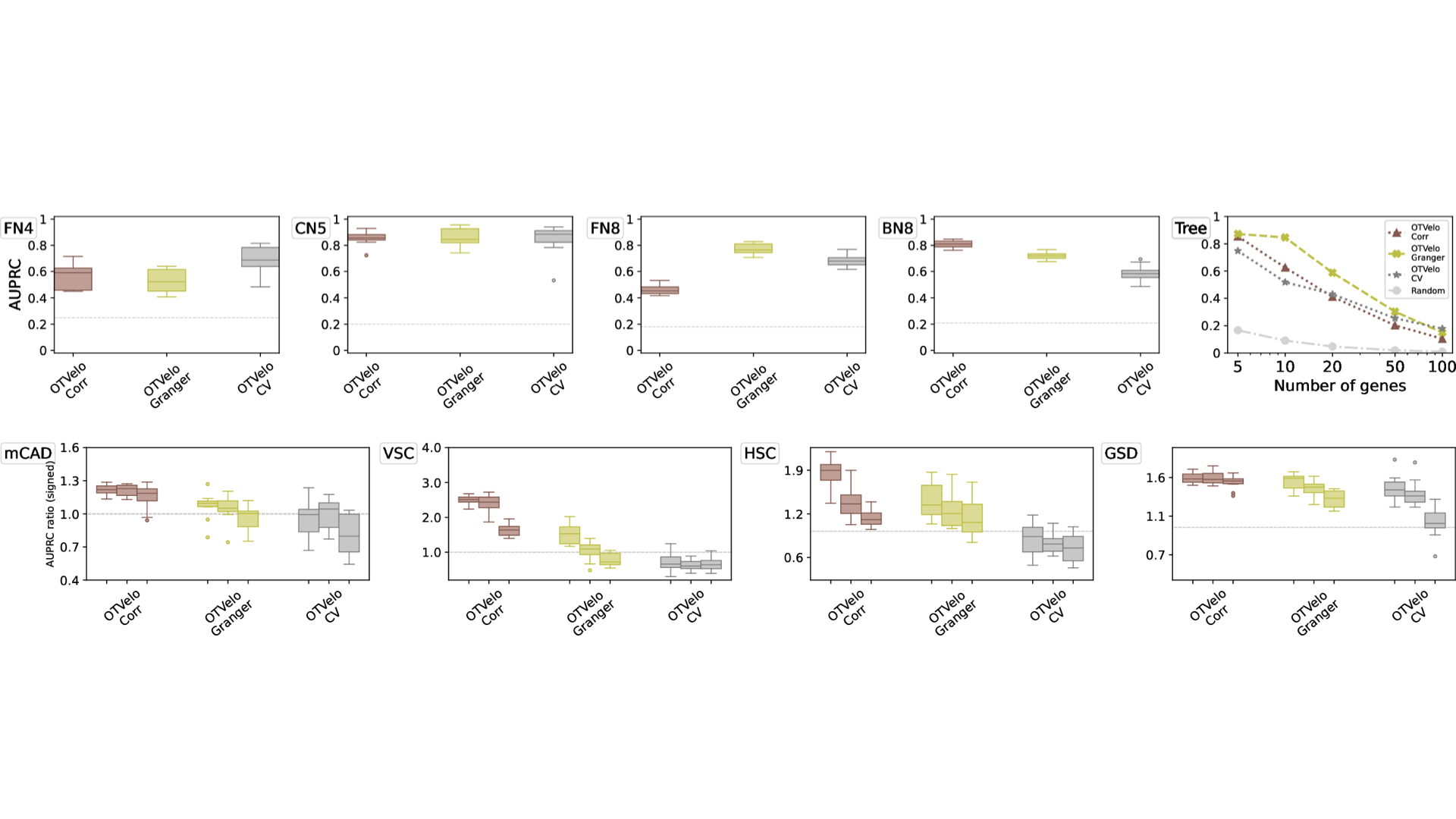

Supplement: S5 Fig — The approach with default parameters r=0.5,λ=1 is labeled ‘OTVelo-Granger’, and ‘OTVelo-CV’ indicates a strategy of picking (r,λ) via a 5-fold cross validation over a grid r∈{0,0.5,1.0} and λ∈{0.1,0.4,0.7,1.0,1.3,1.6}, the same grid as in Figs S3 Fig and S4 Fig. (TIF) [file pcbi.1012476.s005.tif]

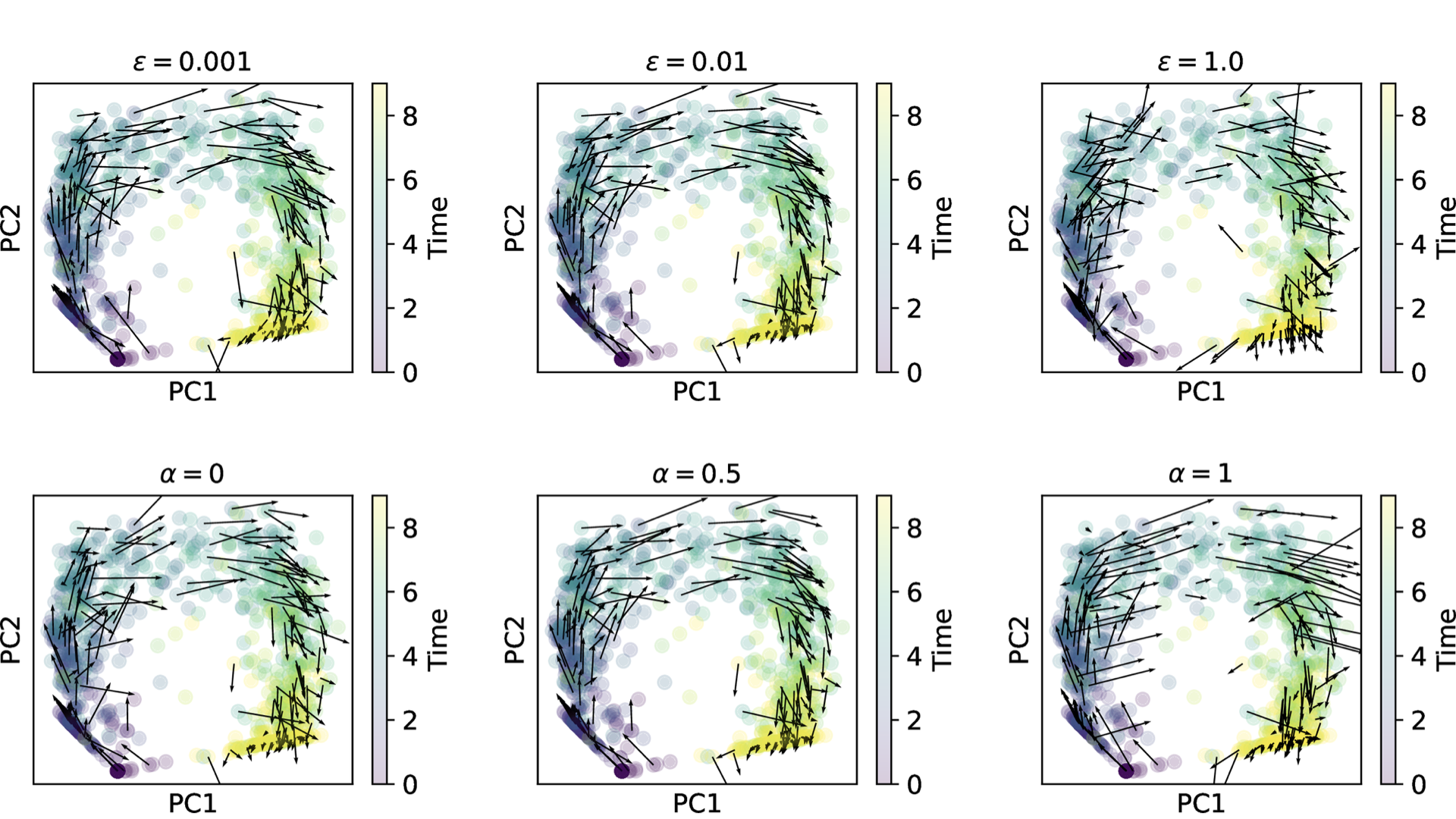

Supplement: S6 Fig — Top: velocity field with α=0.5 and different ϵ. Lower values of ϵ gives velocity fields of good smoothness but can take significant computational time or even fail to converge when ϵ<0.001, while bigger value of ϵ results in non-smooth velocity field. Bottom: velocity field with fixed ϵ=0.01 but different α. α=0 indicates that one only uses OT cost as in Eq (1), while α=1 indicates pure Gromov–Wasserstein OT that penalizes the change in global structure and is less fine-grained. We show that Gromov–Wasserstein appears to smooth out the velocity field too much while OT can be too restrictive, hence a linear combination of both (α=0.5) can yield a smoother velocity field compared to α=0 or α=1. (TIF) [file pcbi.1012476.s006.tif]

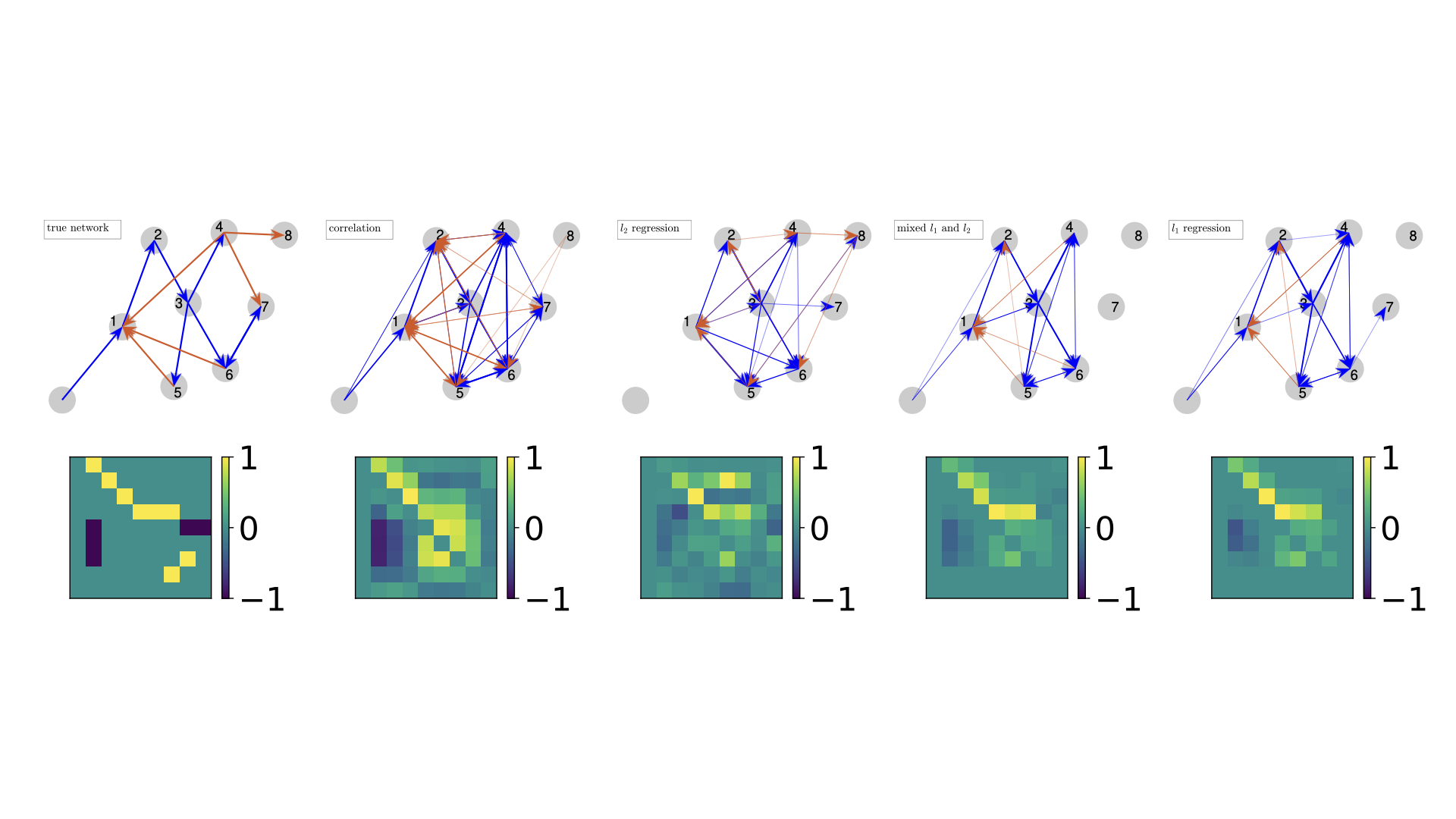

Supplement: S7 Fig — Top: resulting graphs from different approaches. Bottom: weight matrices used to construct the graphs. The correlation approach has default parameter (α,ϵ)=(0.5,0.01), while the regression approaches all have λ=1, and r=0,0.5,1.0 respectively. While the correlation was able to capture most of the structure, the two approaches with l1 regression were able to further reduce the density of graph. (TIF) [file pcbi.1012476.s007.tif]

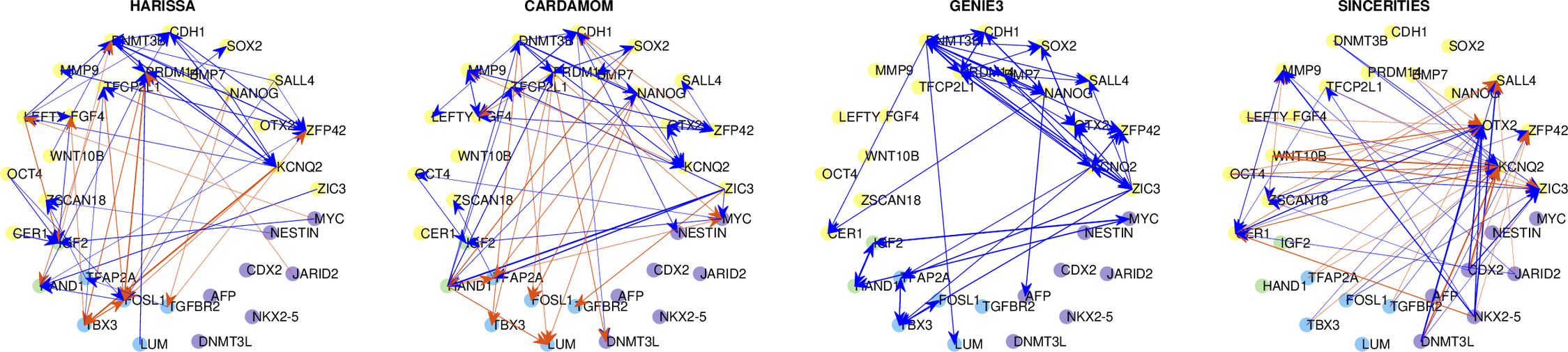

Supplement: S8 Fig — Results of HARISSA, CARDAMOM, GENIE3, and SINCERITIES on scGEM dataset [34], with identical layout as Fig 10. GENIE3 does not identify the type of regulation and all edges are visualized in blue. (TIF) [file pcbi.1012476.s008.tif]

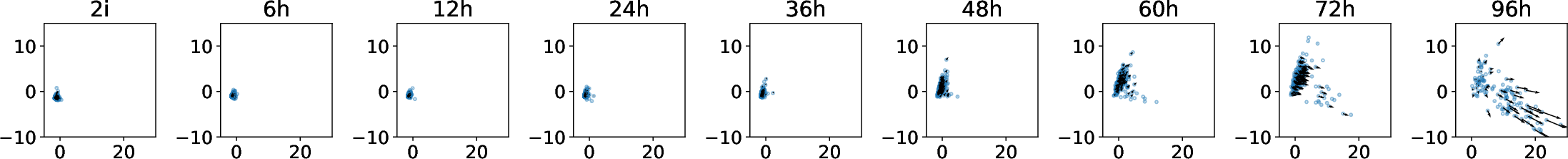

Supplement: S9 Fig — First two principal components of mouse data according to time and the velocity field identified by optimal transport. (TIF) [file pcbi.1012476.s009.tif]

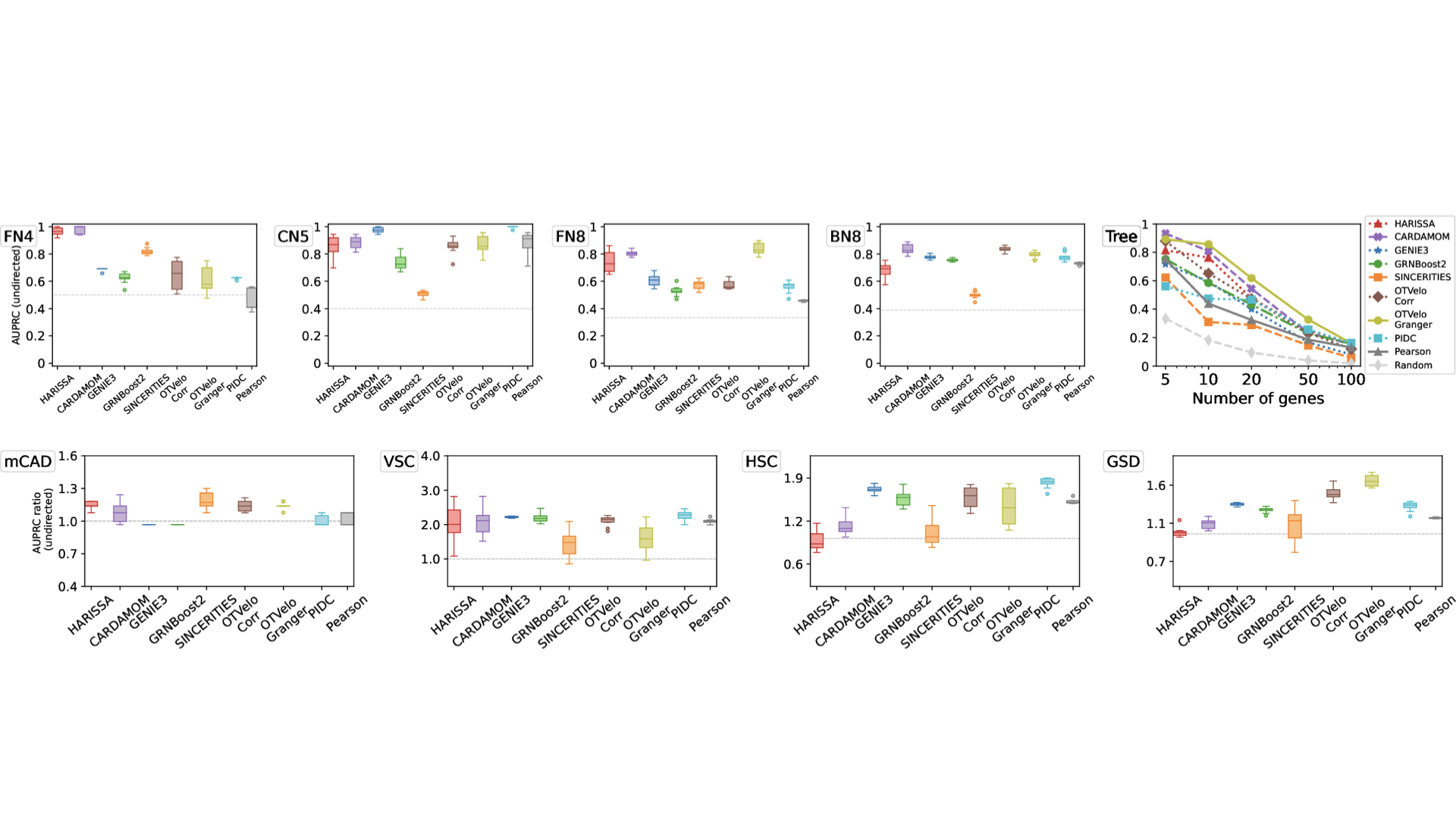

Supplement: S10 Fig — Both ground truth and prediction are symmetrized with edge weights determined by the maximum of absolute value in either directions. (TIF) [file pcbi.1012476.s010.tif]
