# Supplementary material for: Optimal transport reveals dynamic gene regulatory networks via gene velocity estimation
Source: PLoS Comput Biol. 2025 May 8;21(5):e1012476. doi: 10.1371/journal.pcbi.1012476 (PMC12118989; doi:10.1371/journal.pcbi.1012476)
Supplement: Attachment — Submitted filename: ptp-response.pdf [file pcbi.1012476.s011.pdf]

Response to PLOS Computational Biology

Title: Optimal transport reveals dynamic gene regulatory networks via gene velocity estimation

Dear Editor,

Thank you for handling the review process of our manuscript. We are grateful to the reviewers for their very careful reading of the manuscript and for providing constructive and helpful suggestions on how we can improve the paper.

We have modified the manuscript according to the reviewers' comments. In particular, we have included new benchmarking results that expand the comparison of methods and utilize real scRNA-seq datasets. Below is a point-by-point response where we italicized our answers.

Thank you for your time and consideration.

Best regards,

Wenjun Zhao, Erica Larschan, Bjorn Sandstede, and Ritambhara Singh

1 Reviewer 1

The manuscript titled "Optimal transport reveals dynamic gene regulatory networks via gene velocity estimation" presents an innovative approach to infer dynamic gene regulatory networks (GRNs) using optimal transport (OT) and gene velocity estimation from time-stamped single-cell RNA sequencing data. The method, OTVelo, integrates optimal transport theory with gene velocity estimates to model gene-to-gene interactions across time points. The authors successfully demonstrate the efficacy and accuracy of OTVelo on simulated, curated, and experimental datasets, offering valuable insights into gene regulatory dynamics during processes such as cell differentiation and reprogramming. The manuscript is well-written and the results are clearly presented, but there are several areas that could benefit from further clarification or elaboration. Below are some revision suggestions that may help improve the manuscript.

1. To improve the manuscript, it is suggested that the quality of the figures be optimized, as some of them appear unclear or low-resolution.

Response: *We thank the reviewer for the suggestion. We replaced all low-resolution images with vector-format or high-resolution figures.*

2. The authors should carefully verify the accuracy of the results presented in Figure 7, particularly the AUPR scores, which are below 0.64. These low scores may raise concerns regarding the model's performance, and ensuring the accuracy of the data could enhance the reliability of the conclusions drawn from the results.

Response: *We verified that the results agree with a prior publication [Ventre et al.(2023)]. We also included a detailed discussion of why the specific dataset is challenging for GRN inference in section 3.3.4, page 19, lines 512-517. To further demonstrate performance,*

we included results for five additional classical benchmarking scRNA-seq datasets in subsection 3.3.1, page 16-17, lines 445-461 and Figure 9, demonstrating the effectiveness of our algorithm compared to other algorithms. We computed the networks according to the three mouse datasets and two human datasets based on the top variable genes and known transcription factors, and the results were compared against ChIP-seq database. OTVelo-Granger outperformed on most datasets as shown in Figure 9.

3. It is suggested that the comparison of methods could be expanded. Including at least SCENIC, a widely used approach in gene regulatory network inference, would make the comparison more persuasive. This addition would provide valuable insights into the strengths and weaknesses of both methods and help highlight the advantages of the proposed approach.

Response: *Thank you for suggesting to include SCENIC [Aibar et al.(2017)] as another benchmarking method to compare with. SCENIC is a tool for network inference and clustering, and its network inference part is based on GENIE3 [Huynh-Thu et al.(2010)] and GRNBoost2 [Moerman et al.(2018)]: both are tree-based regression methods, and GRNBoost2 is a faster alternative to GENIE3. GENIE3 was included in the original manuscript, and we have included GRNBoost2 in our revision in Figures 3, 5, and 9. As expected, GRNBoost2 has similar performance to GENIE3 since they are based on similar regression models. Furthermore, for the prediction of undirected networks, we also included PIDC [Chan et al.(2017)] and Pearson correlation in the comparisons presented in the Supplementary Figure S10.*

4. It is suggested that the authors elaborate on the impact of different parameters (e.g., time lag, number of time points) on the performance of the OTVelo framework, potentially through sensitivity analyses or additional experiments.

Response: *Thank you for this suggestion. We used simulated data generated from HARISSA [Herbach(2023)] to investigate the effects of varying time lag, number of cells, and final time point. The setup of our new experiments and our findings are reported in section 3.2.1, page 12, lines 367-374 and Figure 4.*

A summary of the dependence and sensitivity of results on the choice of hyperparameters (such as entropy regularization) used in the algorithm can be found in section 3.1, page 11, lines 312-325 with the details on robustness of our method for a range of hyperparameters and the resulting statistics supporting our discussion contained in supplementary Figures S3-S7.

5. It is suggested to provide a detailed user manual for the software available on GitHub, including instructions on how to run the program and explanations of the parameters. This would help users better understand the software and facilitate its application in different research settings.

Response: *We thank the reviewer for the suggestion on facilitating its application. We have included a Jupyter notebook as demo for our algorithm* `https://github.com/sandstede-lab/OT-Velocity/blob/main/Tutorial.ipynb`. *More examples can be found in the same GitHub repository, on both simulated and real datasets.*

2 Reviewer 2

This paper introduces OTVelo, a tool that uses fused Gromov Wasserstein optimal transport to estimate gene dynamics from time stamped scRNA-seq data and infer GRN edges using a correlation-based approach and a Granger causality approach. The manuscript is well written with clear description of method details. Open-source codes are provided

with easy-to-follow instructions. OTVelo is compared with several other methods on both synthetic datasets and real datasets. My main concern is a relative lack of comparison and clarification on performance on real datasets, in addition to some minor questions about the setup of the method. Overall, I believe this is a novel approach with promising results and is worth publishing if the performance evaluation on real datasets could be improved.

Specific points:

1. It would be helpful to explore the stability of the inferred GRN with respect to the marginal distributions, which are set to uniform distributions in the current work. Some growth/death is expected in multi-time point scRNA-seq data as the time points are often relatively distant. For example, WaddingtonOT uses additional information to estimate a "growth rate".

Response: *We thank the reviewer for suggesting to utilize non-uniform marginal distributions to account for unbalanced branches. The current framework can be extended to take account of non-uniform marginal distributions by adapting the definition of cell velocity with a minor adjustment in the normalization, and we would highly recommend using a coupling matrix that considers such possibility. Estimating growth/death rates often relies on domain knowledge (specifically, gene signatures for specific genes relevant to growth and death) and can be incorporated if such prior knowledge is available. Since none of the datasets used in this work contains this information, we were not able to test this approach for experimental datasets. Instead, we investigated this approach on data simulated by BEELINE and mimicked the unbalanced setup by downsampling BEELINE data. These experiments and our findings are discussed in section 3.2.2, pages 15-16, lines 420-435 and Figure 8. The new results indicate improved performance after incorporating non-uniform weights, in particular for multi-branched datasets.*

2. Could you also demonstrate how using FGW would improve the trajectory constructed using just Wasserstein as is done in WaddingtonOT? It seems counter-intuitive to me as the GW part would preserve the pairwise distances which might lead to missing branching structures.

Response: *The Wasserstein term aligns data points in gene expression space, but it does not respect local geometry (two nearby expression vectors could be mapped far apart from each other). In contrast, the GW term attempts to preserve neighbor relationships (but not the actual distances in gene expression space since we use knn-distances). Together, they achieve alignment, while also keeping neighboring relationships intact. Since optimal transport preserves the given marginals, fGW cannot ignore branches: assuming balanced cell-type representation across time points, the GW term will ensure geometric integrity of the matched datapoints, while using only the Wasserstein term could lead to overfitting. To validate this point, we make three observations: (1) Figure S6(b) shows that using Wasserstein and GW with equal weights ($\alpha = 0.5$) results in a smoother velocity field compared with using solely Wasserstein ($\alpha = 0$) or solely GW ($\alpha = 1$). (2) Figures S3 and S4 indicate that $\alpha = 0.5$ may achieve better results. (3) We have visualized the trajectories in Figures 3, 5, and 8, with arrow directions computed from each cell and its image under barycentric projection, showing that our algorithm is able to delineate the underlying structure.*

3. Related to the point above, the quality of the inferred GRN highly depends on the accuracy of the reconstructed trajectory. More evaluations of the trajectory part would be helpful. For example, is it sensitive to choice of parameters including the weights in FGW and the strength of entropy regularization.

Response: *In the original version of manuscript, we reported that the performance is*

robust to the weights α in FGW and ϵ in entropy regularization (see Supplementary Figure S4). Good performance is achieved typically for $\alpha = [0.25, 0.5, 0.75]$ and $\varepsilon < 1$. For the revision, we further investigated the effect of simulation parameters, such as time lag and number of time points. The new numerical results are reported in section 3.2.1, page 12-13, lines 367-374 and Figure 4. The main finding is that the performance of our algorithm depends on experimental design, such as the length of the time series and the lag between consecutive time points. Additional visualization of trajectories on simulated data can be found in the second row of Figure 3 and Figure 5 (for balanced datasets) and top row of Figure 8 (for disproportionate datasets), demonstrating that our trajectories are able to recover the underlying dynamics.

4. The better performance of OTVelo on simulated datasets is well demonstrated. However, the comparison to other methods on real data is rather insufficient. I suggest: (1) utilizing the real dataset benchmarks in BEELINE and in a more recent comparison paper: Stock, Marco, et al. "Topological benchmarking of algorithms to infer gene regulatory networks from single-cell RNA-seq data." Bioinformatics (2024): btae267.; (2) include more related methods, such as GRNBoost2; (3) if possible, also evaluate the performance on the task of find undirected edges and compare to methods like PIDC. For (3), I am fine if the authors find undirected edges irrelevant to the focus of the current work. In addition, it would be helpful to elaborate more on the evaluation results. For example, OTVelo outperforms SINCERITIES on signed AUPRC and underperforms SINCERITIES on other metrics including signed AUROC. In what application scenario is signed AUPRC more important than signed AUROC?

Response: *We thank the reviewer for suggesting additional benchmarking methods and real data examples. Here is a point-to-point response to the four suggestions:*

(1) We have included benchmarking results on all five datasets used by BEELINE (three of them were also used in the paper [Stock et al.(2024)] suggested by the reviewer). The new results can be found in section 3.3.1, page 16-17, lines 445-461 and Figure 9: as shown there, our method outperforms on three out of five datasets by comparison against cell-type specific ChIP-seq networks from known databases.

(2) GRNBoost2 has been included for both simulated and real datasets (Figures 3, 5, and 9). Its performance is very similar to GENIE3.

(3) Initially we did not include undirected methods such as PIDC because we focused on predicting directed and signed networks. For the revision, we performed further experiments on undirected prediction and included PIDC [Chan et al.(2017)] in the comparison as suggested: both prediction and ground truth are symmetrized by taking maximum absolute values of weights for both directions between any pair of genes. The results are shown in supplementary Figure S10. For the undirected task, our method achieves best performance on FN8, BN8, tree networks, and GSD, while PIDC, as one of the best undirected prediction methods [Pratapa et al.(2019)], outperforms on CN5, VSC, and HSC. The overall performance is comparable between OTVelo and PIDC, while as a signed and directed method, OTVelo provides richer information about direction and type of causality.

(4) We have included an additional paragraph "performance metrics" in section 3.1, page 10, lines 307-311 in which we discuss the different options of metrics (AUPRC, AUROC, AUPRC ratio, and early precision) and outline what they each measure and which may be preferable given the specific scenario one may be interested in.

5. There is another work that applies OT to GRN inference, though with different approaches. It would be helpful to also survey this method in the introduction: Lamoline, François, et al. "Gene regulatory network inference from single-cell data using optimal

transport." bioRxiv (2024): 2024-05.

Response: *We thank the reviewer for mentioning this line of research. We have expanded the introduction section to survey optimal-transport based algorithms (page 2, lines 50-62), including the paper suggested [Lamoline et al.(2024)] and several others [Guan et al.(2024), Yachimura et al.(2023), Zhang(2024)] that we are aware of.*

3 Reviewer 3

Inferring gene regulatory networks from gene expression data is an important and challenging problem for the systems biology community. The authors proposed OTVelo, a method for inferring gene regulatory relationships using time-stamped single-cell transcriptome data. OTVelo combines optimal transport and Granger causality, and also uses temporal trajectory to infer gene velocity, which is a meaningful and novel attempt. OTVelo shows comparable or slightly better performance than existing algorithms on several simulated and experimental datasets. Yet the authors also showed that the overall performance of all algorithms is not good enough. GRN inference remains a challenging task and deserves further efforts. To improve this paper, further discussion and tests may be needed.

1. According to Fig. 2 and Fig. 3, it seems that in cases where the network is more complex, the advantages of OTVelo would be more obvious. Can the authors further provide rationales behind such a phenomenon?

Response: *Since OTVelo relies on correlations across each pair of consecutive time points, the algorithm performs best when gene expression levels change rapidly between adjacent time samples (which, for a fixed overall number of time samples, is more likely in complex networks with longer regulatory pathways), while it may perform less well in cases where*

expression levels of different genes correlate only across a longer time range (which, as in FN4, may occur when we sample too often in a relatively simple network so that gene expressions levels vary only slowly in time). In contrast, model-based methods such as HARISSA and CARDAMOM may not capture the biologically more intricate dynamics present in the experiment-based BEELINE datasets or can get stuck locally and fail to converge to the global optima for the more complex CARDAMOM datasets. We included a paragraph in our revision (Page 12, lines 360-365).

2. In Section 3.3.1, Fig. 6, and Fig. S8, the accuracy of gene regulatory networks derived by different algorithms should be quantitatively compared using ground-truth. The authors may quantify how well the regulatory relationships identified by the algorithm match the previously reported relationships. For example, identify how well key regulatory genes inferred by algorithms match with transcription factor activity in corresponding ATAC-Seq or ChIP-Seq data.

Response: *We thank the reviewer for suggesting better benchmarking strategies. We added new performance metrics in Table 3, page 18 that summarize comparisons of predictions against cell-type specific ChIP-seq [Hamaneh and Yu(2019)] and the STRING database [Szklarczyk et al.(2018)] and included a paragraph in section 3.3.2, page 18, lines 479-483 with a discussion of the results. As a summary, our correlation approach achieves either best (on STRING network) or second best (on ChIP-seq network) in AUPRC.*

3. In Fig. 7, the overall performance of all algorithms is poor. It may not be necessary to overemphasize OTVelo's slightly higher AUPRC value in line 428. The authors may want to objectively discuss why GRN inference is challenging on these experimental data.

Response: *We agree and included a paragraph in section 3.3.4, page 19, lines 512-517, where we discuss the challenges for this particular dataset. We also changed the title of this*

section to "OTVelo reveals consistent temporal dynamics on mouse data with stimulus" to address this concern.

4. OTVelo appears to be limited to only some specific datasets. Experimentally derived single-cell transcriptome data not only contain cells in different cell cycles, but also different cell types. This may limit the scope of OTVelo. Can appropriate pre-processing, such as removing cell cycle effects and performing cell classification, help improve OTVelo?

Response: *Appropriate pre-processing can indeed enhance GRN inference algorithms. For instance if the network is believed to be context-specific, such as reflect a particular developmental stage or cell type, then first classifying by cell type and taking the corresponding subset of cell population would likely improve performance. In the drosophila datasets in the manuscript, for example, we used only the neuroectodermal cells instead of the whole organism and extracted the gene-regulatory network for this cell-type only.*

Unless the focus is on discovering cell-cycle specific gene regulation, removing cell-cycle effects may improve performance by filtering out periodic signals that may otherwise hide or over-power other gene-regulatory pathways, (for instance, when the focus is on cell-type differentiation). Cell-cycle information can also be incorporated in the trajectory inference step: in Waddington OT [Schiebinger et al.(2019)] for instance, gene signatures of cell cycles are used to learn the growth rate for each cell (so cells proliferating actively can receive higher weights at the next time point).

Cell classification is also useful when cell types are not represented evenly across time points. Knowing the fraction of cells of each type improves prediction by using weighed marginals to address unbalanced representation. We provided numerical results based on simulations in section 3.2.2, pages 15-16, lines 420-435 where we demonstrate improved performance by incorporating nonuniform marginal distributions.

In the paper, we have presented OTVelo as a general GRN inference method. However, as discussed above, with appropriate pre-processing and/or task-specific formulations, it can be easily adapted to the biological question of interest.

5. PCA is a linear dimension reduction method, which works well when the gene dimension is not high. However, when the genetic dimension is increased and the main component is less significant, PCA will have limited performance. For example, in Fig. 9, PCA dimension reduction cannot reflect cell trajectories over time. Non-linear dimensionality reduction methods such as t-SNE or UMAP are recommended.

Response: *We revised Figure 13 on page 22 by switching from PCA to UMAP, which indeed captures the bifurcating structure better.*

6. The authors emphasizes that OTVelo can infer gene velocity and reflect cell velocity on a dimension reduction map. Can the authors compare OTVelo with RNA velocity or methods alike on experimental data?

Response: *We thank the reviewer for this suggestion. None of the datasets we benchmarked on have splicing information to compute RNA velocity. Instead, we utilized the mouse pancreas dataset [Bastidas-Ponce et al.(2019)] used also in scVelo [Bergen et al.(2019)] and compared the OTVelo gene velocities to the RNA velocities calculated using scVelo. As shown in page 6, lines 159-163 and Figure 2, the two methods agree qualitatively reasonably well, though OTVelo does not fully capture the cell cycle in the upper left part on Figure 2 compared to RNA velocity. Quantitative comparisons are difficult to obtain given the different scaling used in these methods. We intend to include RNA velocity (when available) more directly in our fGW algorithm in a follow-up work.*

References

[Aibar et al.(2017)] S. Aibar, C. B. González-Blas, T. Moerman, V. A. Huynh-Thu, H. Imrichova, G. Hulselmans, F. Rambow, J.-C. Marine, P. Geurts, J. Aerts, J. van den Oord, Z. K. Atak, J. Wouters and S. Aerts. Scenic: single-cell regulatory network inference and clustering. *Nature Methods* **14** (2017) 1083–1086.

[Bastidas-Ponce et al.(2019)] A. Bastidas-Ponce, S. Tritschler, L. Dony, K. Scheibner, M. Tarquis-Medina, C. Salinno, S. Schirge, I. Burtscher, A. Böttcher, F. J. Theis, H. Lickert and M. Bakhti. Comprehensive single cell mrna profiling reveals a detailed roadmap for pancreatic endocrinogenesis. *Development* **146** (2019).

[Bergen et al.(2019)] V. Bergen, M. Lange, S. Peidli, F. A. Wolf and F. J. Theis. Generalizing rna velocity to transient cell states through dynamical modeling. *bioRxiv* (2019).

[Chan et al.(2017)] T. E. Chan, M. P. H. Stumpf and A. C. Babtie. Gene regulatory network inference from single-cell data using multivariate information measures. *Cell Syst.* **5** (2017) 251–267.e3.

[Guan et al.(2024)] V. Guan, J. Janssen, H. Rahmani, A. Warren, S. Zhang, E. Robeva and G. Schiebinger. Identifying drift, diffusion, and causal structure from temporal snapshots (2024).

[Hamaneh and Yu(2019)] M. B. Hamaneh and Y.-K. Yu. Exploring induced pluripotency in human fibroblasts via construction, validation, and application of a gene regulatory network. *PLOS ONE* **14** (2019) e0220742.

[Herbach(2023)] U. Herbach. Harissa: Stochastic simulation and inference of gene regulatory networks based on transcriptional bursting. *Lecture Notes in Computer Science* (2023) 97–105.

[Huynh-Thu et al.(2010)] V. A. Huynh-Thu, A. Irrthum, L. Wehenkel and P. Geurts. Inferring regulatory networks from expression data using tree-based methods. *PLOS ONE* **5** (2010) 1–10.

[Lamoline et al.(2024)] F. Lamoline, I. Haasler, J. Karlsson, J. Gonçalves and A. Aalto. Gene regulatory network inference from single-cell data using optimal transport (2024).

[Moerman et al.(2018)] T. Moerman, S. Aibar Santos, C. Bravo González-Blas, J. Simm, Y. Moreau, J. Aerts and S. Aerts. Grnboost2 and arboreto: efficient and scalable inference of gene regulatory networks. *Bioinformatics* **35** (2018) 2159–2161.

[Pratapa et al.(2019)] A. Pratapa, A. P. Jalihal, J. N. Law, A. Bharadwaj and T. M. Murali. Benchmarking algorithms for gene regulatory network inference from single-cell transcriptomic data. *bioRxiv* (2019).

[Schiebinger et al.(2019)] G. Schiebinger, J. Shu, M. Tabaka, B. Cleary, V. Subramanian, A. Solomon, J. Gould, S. Liu, S. Lin, P. Berube, L. Lee, J. Chen, J. Brumbaugh, P. Rigollet, K. Hochedlinger, R. Jaenisch, A. Regev and E. Lander. Optimal-transport analysis of single-cell gene expression identifies developmental trajectories in reprogramming. *Cell* **176** (2019) 928–943.

[Stock et al.(2024)] M. Stock, N. Popp, J. Fiorentino and A. Scialdone. Topological benchmarking of algorithms to infer gene regulatory networks from single-cell rna-seq data. *Bioinformatics* **40** (2024).

[Szklarczyk et al.(2018)] D. Szklarczyk, A. L. Gable, D. Lyon, A. Junge, S. Wyder, J. Huerta-Cepas, M. Simonovic, N. T. Doncheva, J. H. Morris, P. Bork, L. J. Jensen and C. Mering. String v11: protein–protein association networks with increased coverage, supporting functional discovery in genome-wide experimental datasets. *Nucleic Acids Research* **47** (2018) D607–D613.

[Ventre et al.(2023)] E. Ventre, U. Herbach, T. Espinasse, G. Benoit and O. Gandrillon. One model fits all: combining inference and simulation of gene regulatory networks. *bioRxiv* (2023).

[Yachimura et al.(2023)] T. Yachimura, H. Wang, Y. Imoto, M. Yoshida, S. Tasaki, Y. Kojima, Y. Yabuta, M. Saitou and Y. Hiraoka. scegot: Single-cell trajectory inference framework based on entropic gaussian mixture optimal transport. *bioRxiv* (2023).

[Zhang(2024)] S. Y. Zhang. Joint trajectory and network inference via reference fitting (2024).
